# Obstructive sleep apnea, CPAP therapy, and gastroesophageal reflux symptoms: evidence from a prospective cohort study

**DOI:** 10.3389/fneur.2026.1778762

**Published:** 2026-03-17

**Authors:** Zhang Li, Zhang Wo, Du Yuxuan, Zhu Zhiyuan, Hou Zhijuan, Shan Shan

**Affiliations:** 1 Department of the Fourth Deployed Clinics, Bethune International Peace Hospital, Shijiazhuang, Hebei, China; 2 North China University of Science and Technology, Tangshan, Hebei, China; 3 Hebei Medical University, Shijiazhuang, Hebei, China; 4 Department of Otolaryngology Head and Neck Surgery, Bethune International Peace Hospital, Shijiazhuang, Hebei, China

**Keywords:** CPAP therapy, gastroesophageal reflux, GERD-Q, hypoxic burden, obstructive sleep apnea, oxygen desaturation, prospective cohort, T90%

## Abstract

**Background:**

Obstructive sleep apnea (OSA) frequently coexists with gastroesophageal reflux disease (GERD), yet the degree to which hypoxic burden contributes to reflux severity and whether symptoms improve with continuous positive airway pressure (CPAP) remains insufficiently defined. Objective: To investigate the cross-sectional associations of OSA severity and nocturnal hypoxic burden with GERD symptom presence and severity, and to evaluate longitudinal changes in reflux symptoms after CPAP therapy in a prospective follow-up cohort.

**Methods:**

In a prospective cohort of adults undergoing polysomnography, 580 participants were evaluated at baseline and stratified by GERD status using the GERD-Q (≥8 indicating GERD). OSA severity (apnea–hypopnea index, AHI) and nocturnal hypoxemia burden (T90%, minimum SpO₂, oxygen desaturation index) were compared across reflux strata. Multivariable linear and logistic regression models assessed continuous associations between sleep parameters and GERD severity. A CPAP sub-cohort of moderate-to-severe OSA with concomitant reflux (*n* = 150; completed follow-up, *n* = 112) was monitored for GERD-Q changes at 3 and 6 months.

**Results:**

GERD prevalence reached 29.0%. GERD-Q scores demonstrated dose–response escalation across both OSA severity and hypoxemia categories (*p* for trend <0.001). T90% and ODI showed stronger associations with reflux burden than AHI in fully adjusted models (T90% *β* = 0.81; ODI *β* = 0.44; both *p* < 0.001), while minimum SpO₂ remained inversely related (*β* = −0.18; *p* = 0.003). Logistic models confirmed increased GERD risk in moderate (OR = 1.87) and severe OSA (OR = 2.74), and in patients with marked nocturnal hypoxemia (T90 > 10%; OR = 3.42; all *p* < 0.001). CPAP therapy yielded progressive symptom reduction (GERD-Q − 1.92 at 3 months; −3.28 at 6 months), with clinically meaningful improvement in 69.6% at 6 months.

**Conclusion:**

OSA severity and nocturnal desaturation jointly predict reflux symptom burden, with hypoxic load outperforming event frequency. CPAP produced sustained GERD symptom improvement over 6 months.

## Background

Obstructive sleep apnea (OSA) is a prevalent sleep-related breathing disorder, affecting approximately 10–30% of adults worldwide and occurring more frequently in middle-aged and overweight individuals (1–3). The condition is characterized by recurrent upper-airway collapse during sleep, leading to intermittent hypoxemia, marked swings in intrathoracic pressure, and substantial sleep fragmentation (4–7). These pathophysiologic disturbances have been linked to a broad spectrum of cardiovascular, metabolic, and neuropsychiatric conditions, underscoring the systemic impact of OSA on health (8–10).

Gastroesophageal reflux disease (GERD) is another highly common chronic disorder, with global prevalence estimates ranging from 15 to 25% (4, 6, 11). Persistent reflux symptoms can substantially impair quality of life and increase the risk of complications such as erosive esophagitis and Barrett’s esophagus (12, 13). In routine clinical settings, particularly sleep clinics, GERD is commonly encountered among patients with OSA, and individuals often report symptoms attributable to both conditions (7, 14, 15). This frequent coexistence suggests a clinically relevant interaction between sleep-disordered breathing and reflux-related symptom burden, rather than a coincidental overlap.

Several physiologic mechanisms support a potential link between OSA and GERD, including exaggerated negative intrathoracic pressure during obstructive events, reduced lower esophageal sphincter tone, altered autonomic and vagal regulation, and arousal-related changes in swallowing and esophageal clearance (1, 2, 12, 16). These mechanisms may be particularly pronounced during sleep and could preferentially promote nocturnal reflux symptoms. Nevertheless, mechanistic plausibility alone does not clarify how different dimensions of OSA severity translate into clinically meaningful reflux symptoms.

Despite growing interest in the OSA and GERD relationship, existing evidence remains inconsistent and limited by several important gaps. Prior studies have often relied on small or single-center cohorts and have primarily focused on apnea–hypopnea index (AHI) as the sole marker of OSA severity, while rarely incorporating indices of nocturnal hypoxic burden such as oxygen desaturation or cumulative hypoxemia (17–19). As a result, it remains unclear whether respiratory event frequency or hypoxic burden is more strongly associated with reflux symptom severity. In addition, data regarding the distribution of specific GERD symptom subtypes across OSA severity levels are scarce. Continuous positive airway pressure (CPAP) therapy may alleviate reflux symptoms by stabilizing the upper airway, reducing intrathoracic pressure fluctuations, improving oxygenation, and enhancing sleep continuity (6, 20). However, evidence supporting the effect of CPAP on GERD remains limited, and few studies have incorporated prospective follow-up with standardized symptom assessment (21). Moreover, whether CPAP-related symptom changes reach clinically meaningful thresholds over time has not been well established.

Therefore, this study aimed to examine the cross-sectional associations between OSA severity and nocturnal hypoxic burden with the presence, severity, and subtypes of GERD symptoms at baseline using both AHI- and hypoxemia-based classifications, and to evaluate longitudinal changes in reflux symptoms following CPAP therapy among patients with moderate-to-severe OSA and concomitant GERD, with particular emphasis on clinically meaningful improvement during short- and intermediate-term follow-up. By integrating symptom-based reflux assessment, multiple indices of hypoxic burden, and prospective CPAP follow-up, this study addresses important gaps in understanding the severity-dependent and potentially modifiable relationship between OSA and GERD.

## Methods

### Study design and participants

This single-center clinical study comprised two prespecified analyses: (A) a baseline cross-sectional analysis of the association between OSA-related indices and GERD symptoms, and (B) a prospective follow-up analysis evaluating changes in GERD symptoms after CPAP therapy. Between January 2020 and December 2024, consecutive adults undergoing overnight attended polysomnography (PSG) at Bethune International Peace Hospital were screened.

The study protocol was approved by the Institutional Ethics Committee of *Bethune International Peace Hospital*, and written informed consent was obtained from all participants prior to enrollment.

### Participant flow and analytic samples

Of 627 screened participants, 580 with complete PSG parameters (AHI, ODI, T90%, minimum SpO₂) and baseline GERD-Q were included in the baseline analysis. Participants were classified as non-GERD (GERD-Q < 8; *n* = 412) or GERD (GERD-Q ≥ 8; *n* = 168).

Among participants meeting criteria for moderate-to-severe OSA (AHI ≥ 15 events/h) and concomitant reflux symptoms (GERD-Q ≥ 8), 150 initiated CPAP therapy. Of these, 112 were included in the final follow-up analysis because they had device-download adherence data and completed GERD-Q assessments at both 3 and 6 months after CPAP initiation.

Among the 150 participants who initiated CPAP, 38 were not included in the 6-month follow-up analysis due to CPAP intolerance or discontinuation, inadequate adherence or unavailable device-download data, loss to follow-up, and/or missing GERD-Q assessments at 3 and/or 6 months.

### Inclusion criteria and exclusion criteria

Participants were eligible for inclusion if they met all of the following conditions: (1) Age between 18 and 70 years; (2) Completion of a full-night attended PSG with available data on apnea–hypopnea index (AHI), oxygen desaturation index (ODI), time spent with SpO₂ < 90% (T90%), and minimum oxygen saturation; (3) Completion of the GERD-Q; (4) Provision of written informed consent to participate in the study.

Participants were excluded if any of the following conditions were present: (1) Use of proton pump inhibitors (PPIs) or H2-receptor antagonists (H2RAs) within the previous 8 weeks; (2) History of esophageal or gastric surgery; (3) Presence of active peptic ulcer disease, Barrett’s esophagus, or esophageal/gastric malignancy; (4) Severe cardiovascular or respiratory diseases, including New York Heart Association (NYHA) class III–IV heart failure or severe chronic obstructive pulmonary disease; (5) Neurological disorders known to interfere with sleep assessment; (6) Psychiatric disorders or pregnancy; (7) Incomplete clinical or sleep monitoring data, including missing PSG parameters or questionnaire information.

### CPAP follow-up cohort

The prospective follow-up cohort included only patients with moderate-to-severe OSA, defined as an AHI ≥ 15 events/h, who also met the threshold for reflux symptoms with a GERD-Q score ≥ 8. Eligible patients initiated treatment with either auto-adjusting or fixed-pressure CPAP, according to clinical evaluation and device availability. Individuals who had documented CPAP adherence data and completed GERD-Q assessments at both the 3-month and 6-month follow-up visits were included in the final analysis of treatment response.

### Measurements and definitions

#### GERD symptom assessment and timing

GERD symptoms were assessed using the Gastroesophageal Reflux Disease Questionnaire (GERD-Q; score range 0–18). Baseline GERD-Q was collected on the same day as the overnight PSG (prior to any CPAP initiation). For the CPAP follow-up cohort, GERD-Q was re-administered at 3 months (±2 weeks) and 6 months (±2 weeks) after CPAP initiation during routine clinic follow-up visits; when an in-person visit was not feasible, the questionnaire was completed by telephone under standardized instructions.

The GERD-Q includes six items scored on a 0–3 frequency scale (0 days to 4–7 days per week) over the prior 7 days, yielding a total score ranging from 0 to 18, and includes two items reflecting symptom impact (sleep disturbance due to reflux symptoms and need for over-the-counter medication). A reduction of ≥3 points in GERD-Q was prespecified as the minimal clinically important difference (MCID), as this magnitude corresponds to a clinically interpretable decrease in weekly symptom frequency across multiple domains and can reflect meaningful improvement in patient-relevant impact items (e.g., sleep disturbance or OTC use improving by at least one frequency category) (22, 23). This threshold was chosen as a conservative indicator of patient-perceived benefit consistent with the general MCID concept.

#### GERD symptom subtypes

For subtype analyses, GERD symptoms were categorized into three prespecified groups based on GERD-Q item responses: (1) nighttime reflux, defined by reporting nocturnal reflux symptoms (heartburn and/or regurgitation) occurring at night; (2) typical reflux symptoms, defined by the presence of heartburn and/or regurgitation as captured by GERD-Q frequency items; and (3) atypical (extra-esophageal) symptoms, including chronic cough and non-cardiac chest pain, assessed concurrently using the symptom questionnaire and analyzed as a separate subtype group. Participants could contribute to more than one subtype category if multiple symptoms were reported (24, 25).

#### Sleep-disordered breathing parameters

Objective sleep and respiratory parameters were obtained from full-night attended PSG. The AHI represented the number of apneas and hypopneas per hour of sleep. The ODI was defined as the number of ≥3% oxygen desaturation events per hour. The percentage of sleep time spent with oxygen saturation < 90% (T90%) was used as an index of nocturnal hypoxemia. Minimum SpO₂ referred to the lowest oxygen saturation recorded during the sleep period.

#### OSA severity classification

OSA severity was classified according to AHI thresholds commonly applied in clinical practice. An AHI ≤ 5 events per hour was considered normal, values between 5 and 15 indicated mild OSA, values between 15 and 30 reflected moderate OSA, and an AHI ≥ 30 denoted severe OSA.

#### Hypoxemia classification

Nocturnal hypoxemia was classified according to the proportion of sleep time spent with oxygen saturation below 90% (T90%). A T90% value < 5% was considered normal, values between 5 and 10% indicated mild hypoxemia, and values ≥ 10% were categorized as moderate-to-severe hypoxemia.

### CPAP adherence

Adherence to CPAP therapy was extracted from device download data. Adequate adherence was defined as usage of ≥4 h per night on at least 70% of recorded nights. Adherence and GERD-Q assessments were evaluated at 1, 3, and 6 months after CPAP initiation.

CPAP was delivered using auto-adjusting or fixed-pressure devices per clinical evaluation. CPAP usage and adherence were obtained from device download records. Adequate adherence was defined as ≥4 h/night on ≥70% of nights. For follow-up analyses, CPAP use was operationalized as (a) a binary adherence status (adherent vs. non-adherent) and/or (b) average nightly usage hours over the interval preceding each follow-up visit (continuous metric), derived from device downloads.

### Missing data

Missingness was assessed prior to analysis. The overall proportion of missing data was <5%; therefore, no imputation was performed. Missing values occurred primarily in covariates collected via questionnaires (CHEI and/or IPAQ) and lifestyle variables (smoking/alcohol), whereas PSG parameters and GERD-Q outcomes were largely complete for the baseline analysis.

### Polysomnography protocol

All participants underwent a full-night, attended polysomnography (PSG) using a standardized digital monitoring system. The recording montage included electroencephalography, electrooculography, electromyography, electrocardiography, airflow (nasal pressure and thermistor), thoracoabdominal respiratory effort belts, body position sensors, and pulse oximetry. Sleep staging and respiratory event scoring were performed manually by trained technologists in accordance with the American Academy of Sleep Medicine (AASM) guidelines. Apnea, hypopnea, and oxygen desaturation events were defined using standard criteria. The AHI, oxygen desaturation index (ODI), percentage of sleep time with SpO₂ < 90% (T90%), and minimum SpO₂ were extracted for analysis.

### Dietary quality and physical activity

Dietary quality was evaluated using the Chinese Healthy Eating Index (CHEI), a validated tool reflecting adherence to the Chinese dietary guidelines (26). Participants completed the CHEI questionnaire on the day of PSG. Total scores ranged from 0 to 100, with higher scores indicating healthier dietary patterns (27). CHEI was used as a covariate in multivariable analyses to account for potential lifestyle-related confounding.

Physical activity levels were assessed using the International Physical Activity Questionnaire—Short Form (IPAQ-SF) (28). The questionnaire captured the duration and frequency of walking, moderate-intensity, and vigorous-intensity activities during the previous week, and results were converted to metabolic equivalent (MET)-minutes per week (29, 30). Physical activity was categorized into low, moderate, and high levels according to established IPAQ scoring criteria.

### Demographic and clinical characteristics

Demographic and clinical information was collected through structured interviews and electronic medical records at the time of polysomnography. Variables included age, sex, and body mass index (BMI). Lifestyle behaviors were recorded, including current smoking status (yes/no) and alcohol consumption (yes/no). Additional clinical data—such as comorbid hypertension, diabetes, and medication history—were obtained to characterize the study population and adjust for potential confounding in multivariable analyses.

### Primary outcome measures

The primary outcomes of this study focused on the relationship between OSA and GERD at baseline, the severity of reflux symptoms, and longitudinal changes following treatment. Baseline associations were evaluated by comparing the presence versus absence of GERD between OSA severity groups. GERD symptom severity was assessed using the GERD-Q as a continuous measure (31). For the CPAP follow-up cohort, changes in GERD-Q scores from baseline to 3 and 6 months (ΔGERD-Q) were used to quantify treatment response.

### Covariates and rationale

Age, sex, and BMI were included as core covariates because they are established determinants of both OSA severity and GERD symptom burden and may confound their association. Lifestyle factors (smoking and alcohol consumption), dietary quality (CHEI), and physical activity (IPAQ) were included to account for behavioral and metabolic factors plausibly related to reflux symptoms and sleep-disordered breathing and to reduce residual confounding.

### Statistical analysis

Descriptive statistics were used to summarize baseline characteristics. Continuous variables with normal distributions were reported as means ± standard deviations, whereas non-normally distributed variables were expressed as medians with interquartile ranges. Categorical variables were presented as frequencies and percentages. Comparisons between the non-GERD and GERD groups were performed using independent-sample *t*-tests or Mann–Whitney *U* tests for continuous variables and *χ*^2^ or Fisher’s exact tests for categorical variables. Differences in GERD-Q scores across OSA severity categories and T90% strata were examined using one-way ANOVA or the Kruskal–Wallis test, followed by Bonferroni correction for multiple comparisons. Linear trends across ordered categories were evaluated using *p* for trend.

To assess continuous associations between sleep-disordered breathing parameters and GERD severity, multivariable linear regression models were constructed with GERD-Q score as the dependent variable. AHI, ODI, T90%, and minimum SpO₂ were examined as primary predictors. Three hierarchical models were fitted: Model 1 (unadjusted); Model 2 (adjusted for age, sex, and BMI); and Model 3 (further adjusted for dietary quality [CHEI], smoking, alcohol use, and physical activity level [IPAQ]). Regression coefficients (*β*), 95% confidence intervals, and *p*-values were reported. Multicollinearity was assessed using variance inflation factors.

Multivariable logistic regression was used to evaluate the independent association between OSA or hypoxemia severity and the presence of GERD. GERD (yes/no) served as the binary dependent variable, while AHI categories and T90% categories were the main predictors. The same modeling structure (Models 1–3) was applied. Odds ratios (ORs), 95% confidence intervals, and trend *p*-values were reported.

The distribution of GERD symptom subtypes—including nighttime reflux, typical symptoms, and atypical symptoms—across OSA severity levels was compared using the *χ*^2^ test.

For the CPAP follow-up cohort, changes in GERD-Q scores at 3 and 6 months were analyzed using paired t-tests and linear mixed-effects models to account for repeated measures over time. Clinically meaningful improvement was defined as a reduction of ≥3 points on the GERD-Q, and the proportion of patients achieving this threshold (MCID attainment) was calculated (22, 23). Missing data were assessed before analysis. As the proportion of missing values was <5%, no imputation was performed. Sensitivity analyses using alternative MCID thresholds (2, 3, and 4 points) were conducted.

All analyses were two-sided, and statistical significance was defined as *α* = 0.05. Statistical procedures were performed using SPSS version 26.0 and R version 4.2.2.

## Results

### Baseline characteristics of the study participants

A total of 627 patients were screened, and 580 met the eligibility criteria and were included in the baseline cross-sectional analysis. Among them, 168 individuals (29.0%) met the diagnostic threshold for GERD, while 412 were classified as non-GERD. In addition, 150 patients with moderate-to-severe OSA and concomitant reflux symptoms initiated CPAP therapy; of these, 112 completed the 6-month follow-up and were included in the treatment-response analysis.

Baseline demographic, lifestyle, dietary, and sleep characteristics of the 580 participants included in the baseline analysis are summarized in Table 1. The GERD and non-GERD groups were comparable in age, sex distribution, smoking status, alcohol consumption, and physical activity levels (all *p* > 0.05). However, patients with GERD had a significantly higher body mass index than those without GERD (25.06 ± 2.89 vs. 24.18 ± 2.71 kg/m^2^, *p* = 0.004).

**Table 1:** Baseline characteristics of the study participants (non-GERD vs. GERD).

Variables	Non-GERD (*n* = 412)	GERD (*n* = 168)	*p*-value
Demographic characteristics			
Age, years (mean ± SD)	41.27 ± 10.84	42.93 ± 11.12	0.128
Female, *n* (%)	138 (33.5%)	63 (37.5%)	0.352
BMI, kg/m^2^ (mean ± SD)	24.18 ± 2.71	25.06 ± 2.89	0.004
Lifestyle factors			
Current smoking, *n* (%)	47 (11.4%)	23 (13.7%)	0.419
Alcohol consumption, *n* (%)	44 (10.7%)	22 (13.1%)	0.402
Physical activity (IPAQ)			0.281
Low, *n* (%)	181 (43.9%)	82 (48.8%)	
Moderate, *n* (%)	162 (39.3%)	61 (36.3%)	
High, *n* (%)	69 (16.7%)	25 (14.9%)	
CHEI dietary score (mean ± SD)	53.74 ± 19.82	47.56 ± 20.17	0.006
Sleep parameters			
AHI, events/h (median [IQR])	11.30 [5.20–24.80]	17.90 [9.40–32.10]	<0.001
ODI, events/h (median [IQR])	8.70 [3.40–21.20]	14.80 [7.60–28.50]	<0.001
T90%, % (median [IQR])	3.10 [0.80–10.50]	6.80 [2.40–15.70]	<0.001
Minimum SpO₂, % (mean ± SD)	84.92 ± 5.31	82.16 ± 6.04	<0.001

Data are expressed as mean ± standard deviation, median [interquartile range], or number (percentage), as appropriate. *p-*values were derived from independent-sample *t*-tests or Mann–Whitney *U* tests for continuous variables and *χ*^2^ tests for categorical variables.

BMI, body mass index; CHEI, Chinese Healthy Eating Index; IPAQ, International Physical Activity Questionnaire; AHI, apnea–hypopnea index; ODI, oxygen desaturation index; T90%, percentage of total sleep time with oxygen saturation <90%.

Dietary quality assessed by the Chinese Healthy Eating Index (CHEI) was significantly lower in the GERD group (47.56 ± 20.17 vs. 53.74 ± 19.82, *p* = 0.006). Regarding sleep-related parameters, all indices indicated more severe sleep-disordered breathing in patients with GERD, including higher AHI, higher ODI, greater T90%, and lower minimum nocturnal SpO₂ (all *p* < 0.001). Baseline demographic, clinical, GERD-related, and sleep parameters were comparable between CPAP follow-up completers and non-completers (Supplementary Table S1), suggesting no major selection into follow-up.

### GERD symptoms across OSA severity and nocturnal hypoxemia categories

GERD-Q scores increased progressively with higher AHI categories (Table 2). Patients in the control group (AHI ≤ 5) had the lowest symptom burden (mean 6.82 ± 2.41), whereas those with mild OSA showed moderately higher scores (7.94 ± 2.76, *p* = 0.012 vs. control). GERD-Q scores were substantially elevated in the moderate OSA group (9.08 ± 3.14, *p* < 0.001) and reached the highest levels among patients with severe OSA (10.27 ± 3.52, *p* < 0.001). A significant dose–response relationship was observed across AHI categories (*p* for trend < 0.001).

**Table 2:** GERD-Q scores across AHI and T90% categories.

Category	Subgroup	*n*	GERD-Q score (mean ± SD)	*p*-value
OSA severity (AHI categories)				
AHI ≤ 5 (control)	—	146	6.82 ± 2.41	Reference
5–15 (mild OSA)	—	162	7.94 ± 2.76	0.012
15–30 (moderate OSA)	—	138	9.08 ± 3.14	<0.001
>30 (severe OSA)	—	134	10.27 ± 3.52	<0.001
*p* for trend	—	—	—	<0.001
Nocturnal hypoxemia severity (T90% categories)				
T90% < 5%	Normal oxygenation	244	7.24 ± 2.58	Reference
T90% 5–10%	Mild hypoxemia	186	8.63 ± 2.97	0.004
T90% > 10%	Moderate–severe hypoxemia	150	10.12 ± 3.46	<0.001
*p* for trend	—	—	—	<0.001

GERD-Q scores are presented as mean ± standard deviation. *p*-values were derived from one-way ANOVA with Bonferroni-adjusted *post-hoc* comparisons for pairwise testing.

AHI, apnea–hypopnea index; T90%, percentage of total sleep time spent with oxygen saturation <90%.

Both OSA severity and nocturnal hypoxemia demonstrated significant dose–response relationships with GERD severity.

Similarly, GERD-Q scores increased with worsening nocturnal hypoxemia defined by T90%. Individuals with normal oxygenation (T90% < 5%) had a mean GERD-Q score of 7.24 ± 2.58, whereas those with mild hypoxemia (T90% 5–10%) exhibited higher scores (8.63 ± 2.97, *p* = 0.004). Participants with moderate-to-severe hypoxemia (T90% > 10%) demonstrated the greatest symptom burden (10.12 ± 3.46, *p* < 0.001). The linear trend across T90% categories was highly significant (*p* for trend < 0.001).

### Linear regression analysis of associations between sleep parameters and GERD severity

To examine continuous associations, multivariable linear regression models were constructed with GERD-Q score as the dependent variable (Table 3). In the unadjusted model (Model 1), higher AHI, ODI, and T90% were each associated with higher GERD-Q scores, whereas minimum SpO₂ was inversely associated with symptom severity (all *p* < 0.001).

**Table 3:** Linear regression analysis of associations between sleep parameters and GERD-Q scores in the baseline cohort (*n* = 580).

Sleep parameter	Model 1 coefficient (95% CI)	Model 1 *p-*value	Model 2 coefficient (95% CI)	Model 2 *p*-value	Model 3 coefficient (95% CI)	Model 3 *p-*value
AHI (per 10 events/h)	0.62 (0.41, 0.83)	<0.001	0.48 (0.26, 0.70)	<0.001	0.36 (0.14, 0.59)	0.002
ODI (per 10 events/h)	0.74 (0.53, 0.96)	<0.001	0.61 (0.39, 0.84)	<0.001	0.44 (0.20, 0.68)	<0.001
T90% (per 5% increase)	1.12 (0.83, 1.41)	<0.001	0.97 (0.67, 1.27)	<0.001	0.81 (0.50, 1.11)	<0.001
Minimum SpO₂ (per 1% decrease)	−0.28 (−0.39, −0.17)	<0.001	−0.22 (−0.33, −0.11)	<0.001	−0.18 (−0.29, −0.06)	0.003

Model definitions: Model 1: unadjusted. Model 2: adjusted for age, sex, BMI. Model 3: adjusted for age, sex, BMI, CHEI, smoking, alcohol consumption, and IPAQ.

*β* coefficients represent the expected change in GERD-Q score per unit increase in the sleep parameter. Positive coefficients indicate more severe GERD symptoms with increasing OSA-related burden. CI, confidence interval; AHI, apnea–hypopnea index; ODI, oxygen desaturation index; T90%, percentage of sleep time with oxygen saturation <90%; SpO₂, peripheral oxygen saturation.

These associations persisted after adjustment for age, sex, and BMI (Model 2). In the fully adjusted model (Model 3), which additionally accounted for dietary quality (CHEI), smoking status, alcohol consumption, and physical activity, all sleep-related parameters remained independently associated with GERD-Q scores. Notably, T90% (*β* = 0.81, 95% CI: 0.50–1.11) and ODI (β = 0.44, 95% CI: 0.20–0.68) showed the strongest associations, whereas AHI retained a more modest but significant association (*β* = 0.36, 95% CI: 0.14–0.59). Minimum SpO₂ remained negatively associated with GERD-Q scores (*β* = −0.18, 95% CI: −0.29 to −0.06).

### Logistic regression analysis of OSA severity, hypoxemia, and GERD risk

Multivariable logistic regression analyses were performed to evaluate the independent associations between OSA severity, nocturnal hypoxemia, and the presence of GERD (Table 4). Compared with participants with AHI ≤ 5, those with moderate and severe OSA had progressively higher odds of GERD across all models. In the fully adjusted model, the odds ratios were 1.87 (95% CI: 1.14–3.05) for moderate OSA and 2.74 (95% CI: 1.67–4.48) for severe OSA, with a significant dose–response trend (*p* for trend < 0.001).

**Table 4:** Logistic regression analysis of OSA severity, hypoxemia, and GERD risk in the baseline cohort (*n* = 580).

Predictor	Model 1 OR (95% CI)	Model 1 *p*-value	Model 2 OR (95% CI)	Model 2 *p*-value	Model 3 OR (95% CI)	Model 3 *p*-value
OSA severity (AHI categories)						
AHI ≤ 5 (reference)	—	—	—	—	—	—
5–15 (mild OSA)	1.42 (0.94, 2.14)	0.094	1.36 (0.89, 2.08)	0.155	1.28 (0.82, 1.99)	0.277
15–30 (moderate OSA)	2.18 (1.39, 3.43)	0.001	2.04 (1.28, 3.27)	0.003	1.87 (1.14, 3.05)	0.013
>30 (severe OSA)	3.41 (2.18, 5.34)	<0.001	3.12 (1.96, 4.98)	<0.001	2.74 (1.67, 4.48)	<0.001
*p* for trend	—	<0.001	—	<0.001	—	<0.001
Nocturnal hypoxemia (T90% categories)						
<5% (reference)	—	—	—	—	—	—
5–10%	1.96 (1.32, 2.89)	0.001	1.84 (1.22, 2.79)	0.004	1.63 (1.05, 2.52)	0.029
>10%	4.37 (2.90, 6.58)	<0.001	3.98 (2.60, 6.10)	<0.001	3.42 (2.11, 5.55)	<0.001
*p* for trend	—	<0.001	—	<0.001	—	<0.001

Model definitions: Model 1: unadjusted; Model 2: adjusted for age, sex, BMI; Model 3: adjusted for age, sex, BMI, CHEI, smoking, alcohol consumption, and IPAQ.

Odds ratios (ORs) represent the likelihood of having GERD (GERD-Q ≥ 8) across different levels of OSA or nocturnal hypoxemia severity. AHI, apnea–hypopnea index; T90%, percentage of sleep time with oxygen saturation <90%; CI, confidence interval.

Hypoxemia-based classification showed even stronger associations. Compared with T90% < 5%, participants with T90% > 10% had an adjusted odds ratio of 3.42 (95% CI: 2.11–5.55) for GERD. A highly significant linear trend was observed across T90% categories (*p* for trend < 0.001).

### CPAP treatment effects on GERD symptoms over 6 months

The following analyses focus on longitudinal changes in GERD symptoms after CPAP therapy. Among the 112 patients who completed the 6-month CPAP follow-up, GERD-Q scores declined significantly over time (Table 5).

**Table 5:** Changes in GERD-Q scores after 3 and 6 months of CPAP therapy among patients completing follow-up (*n* = 112).

Time point	GERD-Q score (mean ± SD)	ΔGERD-Q vs baseline	*p*-value	MCID achievement (≥3-point reduction), *n* (%)
Baseline	10.84 ± 3.12	—	—	—
3 months	8.92 ± 2.98	−1.92 ± 2.41	0.001	47 (42.0%)
6 months	7.56 ± 2.84	−3.28 ± 2.67	<0.001	78 (69.6%)

Data are presented as mean ± standard deviation or number (percentage).

*p*-values were derived from paired *t*-tests comparing follow-up scores with baseline values.

MCID was defined as a reduction of ≥3 points in GERD-Q score from baseline.

CPAP, continuous positive airway pressure; GERD-Q, Gastroesophageal Reflux Disease Questionnaire.

The mean GERD-Q score decreased from 10.84 ± 3.12 at baseline to 8.92 ± 2.98 at 3 months (mean change −1.92 ± 2.41; *p* = 0.001), and further declined to 7.56 ± 2.84 at 6 months (mean change −3.28 ± 2.67; *p* < 0.001).

Clinically meaningful improvement, defined as a ≥ 3-point reduction in GERD-Q score, was achieved by 42.0% of patients at 3 months and 69.6% at 6 months. Additional CPAP-related outcomes, including sensitivity analyses using alternative MCID thresholds, are summarized in Supplementary Tables S2–S4 and showed consistent results. In addition, CPAP adherence was modeled as a continuous variable using average nightly usage hours. Greater CPAP use was associated with larger reductions in GERD-Q scores over 6 months (Supplementary Tables S5, S6), supporting a dose–response relationship.

### Distribution of GERD symptom subtypes across OSA severity categories

The distribution of GERD symptom subtypes differed significantly across OSA severity categories (Table 6). Nighttime reflux increased progressively with OSA severity, rising from 13.64% in participants with AHI ≤ 5 to 42.41% in those with severe OSA (*χ*^2^ = 38.27, *p* < 0.001). A similar pattern was observed for typical reflux symptoms (heartburn/regurgitation), which increased from 18.18 to 50.00% across the same categories (*χ*^2^ = 44.86, p < 0.001). In contrast, atypical GERD symptoms, including chronic cough and non-cardiac chest pain, did not differ significantly across OSA severity categories (*χ*^2^ = 5.82, *p* = 0.121).

**Table 6:** Distribution of GERD subtypes across OSA severity categories.

GERD subtype	AHI ≤ 5 (*n* = 132)	Mild OSA 5–15 (*n* = 148)	Moderate OSA 15–30 (*n* = 142)	Severe OSA >30 (*n* = 158)	*χ* ^2^	*p*-value
Nighttime reflux	18 (13.64%)	29 (19.59%)	41 (28.87%)	67 (42.41%)	38.27	<0.001
Regurgitation/heartburn	24 (18.18%)	36 (24.32%)	52 (36.62%)	79 (50.00%)	44.86	<0.001
Atypical symptoms (cough, chest pain)	21 (15.91%)	27 (18.24%)	32 (22.54%)	39 (24.68%)	5.82	0.121

Values are presented as number (percentage).

*χ*^2^ tests were used to compare subtype distribution across OSA severity categories.

Nighttime reflux and regurgitation/heartburn showed a significant increasing trend with higher AHI severity, whereas atypical symptoms did not differ significantly across OSA categories.

AHI, apnea–hypopnea index; OSA, obstructive sleep apnea.

## Discussion

This study provides an integrated assessment of the association between OSA and gastroesophageal reflux symptoms in a large clinical cohort. Several key observations emerged. First, GERD-Q-defined reflux symptoms were more prevalent among patients with OSA, particularly those with moderate-to-severe disease. GERD-Q scores increased progressively across both AHI- and T90%-based severity strata, demonstrating clear dose–response relationships. In multivariable analyses, indices of nocturnal hypoxemia, including T90% and minimum oxygen saturation, were more strongly associated with reflux symptom severity than AHI, suggesting that cumulative hypoxic burden may capture clinically relevant dimensions of sleep-disordered breathing in relation to reflux symptoms. In addition, nighttime reflux and typical reflux symptoms were more frequently reported among patients with severe OSA.

In the prospective CPAP follow-up component, reflux symptom scores declined over time among patients who completed therapy, with larger reductions observed at 6 months and more than 60% achieving a clinically meaningful improvement. These longitudinal changes indicate a reduction in reflux symptom burden during follow-up; however, given the observational nature of the follow-up analysis and the absence of a parallel control group, these findings should be interpreted as treatment-associated symptom changes rather than evidence of a causal effect. Taken together, the present results help characterize severity-dependent associations between OSA-related indices and reflux symptom burden, and suggest that CPAP use may be associated with symptom improvement in selected patients with concomitant OSA and GERD.

The frequent co-occurrence of obstructive sleep apnea and gastroesophageal reflux observed in the present cohort is consistent with previous reports (4, 7, 9, 14, 32–36). However, the current study extends prior work by leveraging a larger clinical sample and by incorporating severity-based analyses that consider both respiratory event frequency and hypoxemic burden. The particularly high prevalence of GERD in severe OSA underscores the need for routine assessment of reflux symptoms in sleep clinic settings (37). This association carries clinical relevance, as reflux may exacerbate OSA through airway mucosal irritation and cough-related arousals, while OSA can in turn promote reflux via intrathoracic pressure swings and altered vagal regulation (1–3, 38). These bidirectional effects suggest a potentially self-reinforcing cycle, highlighting the importance of recognizing and managing both conditions concurrently (35, 39–41).

A notable finding of this study is that markers of hypoxic burden—particularly T90% and minimum oxygen saturation—demonstrated stronger associations with GERD than the apnea–hypopnea index. AHI quantifies the number of respiratory events but does not capture their duration or the cumulative physiological stress they impose (42–44). Measures such as T90% better reflect the sustained intrathoracic pressure swings and prolonged respiratory effort that may promote reflux episodes (45, 46). Intermittent hypoxemia has also been shown to impair vagal regulation and lower esophageal sphincter tone, mechanisms that could facilitate reflux (47, 48). Furthermore, hypoxia-related inflammation and oxidative stress may heighten esophageal sensitivity, while sleep fragmentation linked to hypoxemia can alter swallowing patterns in ways that favor reflux (49–51). Together, these pathways suggest that hypoxic burden may represent a key pathophysiologic feature underlying the OSA–GERD relationship.

In this study, GERD-Q–defined reflux symptoms were more prevalent among patients with OSA and increased in a dose–response manner across both AHI- and hypoxemia-based severity strata. In multivariable analyses, indices of nocturnal hypoxemia, including T90% and minimum oxygen saturation, were more strongly associated with reflux symptom severity than AHI. These findings reflect observational associations and do not establish causality. Nevertheless, several pathophysiological considerations may help contextualize the observed patterns. Compared with AHI, which primarily reflects event frequency, measures of hypoxic burden capture the cumulative physiological stress of sleep-disordered breathing, including sustained intrathoracic pressure fluctuations and prolonged respiratory effort, which may be linked to reflux symptoms through altered lower esophageal sphincter function, autonomic regulation, and esophageal sensitivity.

In this study, nighttime reflux and typical symptoms became increasingly common with greater OSA severity, a pattern consistent with the influence of supine sleep position, amplified intrathoracic pressure swings, and more pronounced upper airway collapse during sleep. These factors may facilitate upward movement of gastric contents and intensify typical reflux manifestations (43). In contrast, atypical symptoms did not vary significantly across OSA categories. Such findings may reflect the subjective nature of these symptoms, their weaker direct linkage to respiratory events, or the need for more objective assessment tools—such as esophageal manometry or 24-h pH impedance monitoring—to capture subtle physiologic differences. The divergent patterns observed across subtypes highlight that not all reflux-related symptoms respond similarly to sleep-disordered breathing burden (47, 52).

In the prospective follow-up component, GERD-Q scores declined significantly over time among patients who completed CPAP therapy, with greater reductions observed at 6 months and more than 60% achieving a clinically meaningful improvement. These findings indicate an improvement in reflux symptom burden during follow-up. However, given the observational nature of this analysis and the absence of a parallel untreated control group, these changes should be interpreted cautiously and cannot be attributed exclusively to CPAP therapy. Several mechanisms may nonetheless help contextualize the observed symptom improvements. By stabilizing the upper airway, CPAP may attenuate intrathoracic pressure swings that facilitate reflux (53, 54). Improvements in sleep continuity could reduce microarousals and associated fluctuations in esophageal pressure, while enhanced nocturnal oxygenation may support autonomic balance and lower esophageal sphincter function (6, 8). Enhanced oxygenation may also restore vagal balance and support lower esophageal sphincter function. Together, these effects can reduce nocturnal swallowing and reflux events (40, 55). These results suggest that CPAP provides dual advantages for patients with OSA and concomitant GERD by addressing both respiratory disturbances and reflux symptoms (56, 57). It should also be noted that the follow-up cohort consisted of patients who initiated and adhered to CPAP and completed longitudinal assessments, raising the possibility of selection effects related to treatment tolerance, adherence, and follow-up completion. Therefore, the observed improvements likely reflect treatment-associated changes in a selected population rather than definitive causal effects.

Although GERD-Q is validated for symptom screening, it cannot differentiate acid reflux, weakly acidic reflux, or non–reflux-related symptoms, and some degree of symptom misclassification is therefore possible. Accordingly, the present findings should be interpreted as associations with reflux symptom burden rather than objectively defined reflux phenotypes. This limitation may have particularly influenced analyses of symptom subtypes. Typical reflux symptoms, which are more specific to reflux physiology, showed clear severity-dependent associations with OSA, whereas atypical symptoms did not. The lack of significant differences for atypical symptoms across OSA severity strata may reflect the limited specificity of symptom-based assessment rather than a true absence of association. Future studies incorporating objective reflux measurements are warranted to further clarify these relationships.

Several methodological limitations should be acknowledged. First, this was a single-center study, and the study population may not fully represent broader or community-based cohorts, potentially limiting generalizability. Second, GERD was assessed using the symptom-based GERD-Q rather than objective diagnostic modalities such as 24-h pH impedance monitoring, and data on esophageal motility and lower esophageal sphincter function were unavailable. In addition, reliance on a symptom-based questionnaire may have introduced misclassification of reflux status, as GERD-Q cannot distinguish acid reflux from weakly acidic reflux or non-reflux-related symptoms. Consequently, the observed associations pertain to reflux symptom burden rather than objectively confirmed gastroesophageal reflux. Third, postural reflux and detailed dietary intake were not systematically evaluated, limiting assessment of positional and lifestyle-related influences. In addition, the prospective CPAP follow-up was observational, subject to loss to follow-up, and restricted to patients who completed treatment and follow-up assessments, raising the possibility of selection bias. The follow-up duration was also limited to 6 months, precluding assessment of long-term durability of symptom changes. Despite comprehensive adjustment, residual confounding from unmeasured or imperfectly measured factors remains possible. Variables related to sleep posture, esophageal anatomy, medication use, and psychosocial status were not captured in the present study and may contribute to the observed associations. Accordingly, the multivariable models were designed to account for major confounders rather than to provide a fully explanatory framework for the OSA–GERD relationship.

Future multicenter longitudinal studies incorporating objective reflux measurements, esophageal functional testing, and controlled evaluations of combined therapeutic strategies—such as CPAP alongside pharmacologic treatment—are warranted to clarify underlying mechanisms and optimize management of patients with coexisting OSA and GERD.

## Conclusion

In conclusion, OSA was associated with GERD symptom burden in a clear severity-dependent manner, with typical reflux symptoms more frequently reported among patients with severe OSA. Measures of hypoxemia-related burden appeared clinically relevant in characterizing these associations. In addition, GERD symptoms declined during follow-up among patients who completed CPAP therapy, suggesting a treatment-associated reduction in symptom burden in selected patients with coexisting OSA and GERD.

## Data Availability

The original contributions presented in the study are included in the article/Supplementary material, further inquiries can be directed to the corresponding author.
