# Supplementary material for: Obstructive sleep apnea, CPAP therapy, and gastroesophageal reflux symptoms: evidence from a prospective cohort study
Source: Front Neurol. 2026 Mar 17;17:1778762. doi: 10.3389/fneur.2026.1778762 (PMC13035730; doi:10.3389/fneur.2026.1778762)
Supplement: Supplementary file 1 [file Table_1.docx]

**Supplementary Table S1. Baseline Characteristics of CPAP Follow-up Completers and Non-completers**

| **Variables** | **Completers (n = 112)** | **Non-completers (n = 38)** | **P value** |
| --- | --- | --- | --- |
| **Demographic characteristics** |  |  |  |
| Age, years (mean ± SD) | 43.68 ± 10.42 | 44.91 ± 11.06 | 0.512 |
| Female, n (%) | 41 (36.61) | 15 (39.47) | 0.742 |
| BMI, kg/m² (mean ± SD) | 25.84 ± 2.91 | 26.12 ± 3.08 | 0.598 |
| **Lifestyle factors** |  |  |  |
| Current smoking, n (%) | 17 (15.18) | 7 (18.42) | 0.638 |
| Alcohol consumption, n (%) | 16 (14.29) | 6 (15.79) | 0.817 |
| **GERD symptom burden** |  |  |  |
| Baseline GERD-Q score (mean ± SD) | 10.86 ± 3.09 | 10.41 ± 3.22 | 0.462 |
| **Sleep-related parameters** |  |  |  |
| AHI, events/h (median [IQR]) | 29.60 [21.30–38.70] | 30.90 [22.40–40.10] | 0.548 |
| ODI, events/h (median [IQR]) | 24.80 [16.90–34.20] | 26.10 [17.80–36.40] | 0.503 |
| T90%, % (median [IQR]) | 14.30 [8.10–22.60] | 15.10 [8.90–23.40] | 0.614 |
| Minimum SpO₂, % (mean ± SD) | 80.92 ± 5.81 | 80.17 ± 6.09 | 0.489 |

Data are presented as mean ± standard deviation, median [interquartile range], or number (percentage), as appropriate.
Comparisons between CPAP follow-up completers and non-completers were performed using independent-sample *t* tests or Mann–Whitney *U* tests for continuous variables and χ² tests for categorical variables.
P values are descriptive and were not adjusted for multiple comparisons.

Abbreviations:
BMI = body mass index; GERD-Q = Gastroesophageal Reflux Disease Questionnaire;
AHI = apnea–hypopnea index; ODI = oxygen desaturation index;
T90% = percentage of total sleep time with oxygen saturation < 90%;
SpO₂ = peripheral oxygen saturation.

**Supplementary Table S2. CPAP Adherence Over 6 Months Among Patients Completing Follow-up (n = 112)**

| **Time Point** | **Average CPAP Use (h/night, mean ± SD)** | **Adherence ≥4 h/night, n (%)** | **Residual AHI (mean ± SD)** |
| --- | --- | --- | --- |
| 1 month | 4.68 ± 1.92 | 67 (59.8%) | 6.42 ± 2.51 |
| 3 months | 5.12 ± 1.84 | 76 (67.9%) | 5.98 ± 2.38 |
| 6 months | 5.38 ± 1.78 | 82 (73.2%) | 5.61 ± 2.21 |

**Notes:**
Values represent device-recorded CPAP usage. Adherence defined as ≥4 h/night.

**Supplementary Table S3. Linear Mixed-Effects Model (LMM) for GERD-Q Changes Over Time Under CPAP Therapy**

| **Fixed Effect** | **β Coefficient (95% CI)** | **P value** |
| --- | --- | --- |
| Time (3 months vs baseline) | −1.94 (−2.41, −1.47) | <0.001 |
| Time (6 months vs baseline) | −3.29 (−3.81, −2.77) | <0.001 |
| Age | 0.02 (−0.01, 0.06) | 0.214 |
| Sex (male = 1) | 0.18 (−0.34, 0.71) | 0.492 |
| BMI | 0.08 (0.01, 0.15) | 0.032 |
| CPAP adherence (h/night) | −0.31 (−0.47, −0.15) | <0.001 |

**Random Effects:**

- Between-subject variance: 1.04
- Residual variance: 0.92

**Notes:**
LMM uses a random intercept per participant. Time modeled categorically. CPAP adherence included as a time-varying covariate.

**Supplementary Table S4. Baseline vs Follow-up Sleep Parameters Under CPAP Therapy (n = 112)**

| **Parameter** | **Baseline (mean ± SD)** | **3 months** | **6 months** | **P value (baseline vs 6 months)** |
| --- | --- | --- | --- | --- |
| AHI (events/h) | 32.46 ± 12.18 | 8.92 ± 4.76 | 7.84 ± 4.51 | <0.001 |
| ODI (events/h) | 28.14 ± 11.32 | 8.11 ± 4.38 | 7.46 ± 4.12 | <0.001 |
| Minimum SpO₂ (%) | 76.52 ± 6.48 | 84.16 ± 5.72 | 85.31 ± 5.61 | <0.001 |
| T90% (%) | 12.88 ± 7.42 | 4.72 ± 3.88 | 3.96 ± 3.54 | <0.001 |

**Notes:**
All parameters showed significant improvement after CPAP therapy.

**Supplementary Table S5. MCID Sensitivity Analysis Using Different Cut-off Definitions**

| **MCID Definition** | **3-Month MCID, n (%)** | **6-Month MCID, n (%)** |
| --- | --- | --- |
| ≥2-point reduction | 59 (52.7%) | 89 (79.5%) |
| ≥3-point reduction (primary) | 47 (42.0%) | 78 (69.6%) |
| ≥4-point reduction | 33 (29.5%) | 61 (54.5%) |

**Notes:**
Across all MCID thresholds, GERD improvement remained consistent and clinically meaningful.

**Supplementary Table S6. Association Between Average Nightly CPAP Use and Change in GERD-Q Score Over 6 Months**

| **Predictor** | **β (95% CI)** | **P value** |
| --- | --- | --- |
| **Average nightly CPAP use, hours** | −0.47 (−0.81, −0.13) | 0.007 |
| **Age, years** | −0.02 (−0.05, 0.01) | 0.186 |
| **Female sex** | −0.31 (−0.88, 0.26) | 0.281 |
| **BMI, kg/m²** | 0.09 (−0.03, 0.21) | 0.134 |
| **Baseline GERD-Q score** | −0.42 (−0.58, −0.26) | <0.001 |

Linear regression analysis was performed to examine the association between CPAP adherence modeled as a continuous variable and change in GERD-Q score over 6 months among patients who completed follow-up (n = 112).
The dependent variable was the change in GERD-Q score from baseline to 6 months (ΔGERD-Q), with negative values indicating symptom improvement.
The primary independent variable was average nightly CPAP use (hours), derived from device download data.
Models were adjusted for age, sex, body mass index (BMI), and baseline GERD-Q score.

β coefficients represent the expected change in ΔGERD-Q score per 1-hour increase in average nightly CPAP use.
P values are two-sided and were not adjusted for multiple comparisons.

Abbreviations:
BMI = body mass index; CPAP = continuous positive airway pressure;
GERD-Q = Gastroesophageal Reflux Disease Questionnaire.
